# Unveiling the Interplay Between Dendritic Cells and Natural Killer Cells as Key Players in *Leishmania* Infection

**DOI:** 10.1155/jimr/3176927

**Published:** 2025-02-10

**Authors:** Ana Valério-Bolas, Mafalda Meunier, Armanda Rodrigues, Joana Palma-Marques, Rui Ferreira, Inês Cardoso, Lis Lobo, Marta Monteiro, Telmo Nunes, Ana Armada, Wilson T. Antunes, Graça Alexandre-Pires, Isabel Pereira da Fonseca, Gabriela Santos-Gomes

**Affiliations:** ^1^Unit for Teaching and Research in Medical Parasitology, Global Health and Tropical Medicine, GHTM, Associate Laboratory in Translation and Innovation Towards Global Health, LA-REAL, Instituto de Higiene e Medicina Tropical, IHMT, Universidade Nova de Lisboa, UNL, Lisbon 1349-008, Portugal; ^2^BSA, Banco de Sangue Animal, Porto 4100-462, Portugal; ^3^Faculty of Veterinary Medicine, Centre for Interdisciplinary Research in Animal Health, CIISA, University of Lisbon, Av. Universidade Técnica, Lisbon 1300-477, Portugal; ^4^Microscopy Center, Faculty of Sciences of the University of Lisbon-FCUL—BioISI Ce3CE, Lisboa, Portugal; ^5^Instituto Universitário Militar (IUM), Centro de Investigação, Desenvolvimento e Inovação da Academia Militar (CINAMIL), Unidade Militar Laboratorial de Defesa Biológica e Química (UMLDBQ), Lisboa 1849-012, Portugal; ^6^Associate Laboratory for Animal and Veterinary Sciences (AL4AnimalS), Lisbon, Portugal

## Abstract

Leishmaniasis is a group of parasitic diseases whose etiological agent is the protozoa *Leishmania*. These diseases afflict impoverished populations in tropical and subtropical regions and affect wild and domestic animals. Canine leishmaniasis is a global disease mostly caused by *L. infantum*. Dogs are recognized as a good reservoir since harbor the infection long before developing the disease, facilitating parasite transmission. Furthermore, there is growing evidence that dogs may also be the reservoir of the American *Leishmania* spp. as *L. amazonensis*. The innate immune response is the first defense line against pathogens, which includes natural killer (NK) and dendritic cells (DCs). By recognizing and ultimately destroying infected cells, and by secreting immune mediators that favor inflammatory microenvironments, NK cells take the lead in the infectious process. When interacting with *Leishmania* parasites, DCs become activated and play a key role in driving the host immune response. While activated DCs can modulate NK cell activity, *Leishmania* parasites can directly activate NK cells by interacting with innate immune receptors. Once activated, NK cells can engage in a bidirectional interplay with DCs. However, the complexity of these interactions during *Leishmania* infection makes it challenging to fully understand the underlying processes. To further explore this, the present study investigated the dynamic interplay established between monocyte-derived DCs (moDCs) and putative NK (pNK) cells of dogs during *Leishmania* infection. Findings indicate that the crosstalk between moDCs exposed to *L. infantum* or *L. amazonensis* and pNK cells enhances chemokine upregulation, potentially attracting other leukocytes to the site of infection. pNK cells activated by *L. infantum* infected DCs upregulate *IL-10*, which can lead to a regulatory immune response while moDCs exposed to *L. amazonensis* induced pNK cells to overexpress *IFN-γ* and *IL-13*, favoring a mix of pro- and anti-inflammatory response. In addition, parasite-derived extracellular vesicles (EVs) can modulate the host immune response by stimulating the upregulation of anti-inflammatory cytokines and perforin release, which may impact infection outcomes. Thus, *Leishmania* and parasitic EVs can influence the bidirectional interplay between canine NK cells and DCs.

## 1. Introduction

Leishmaniasis is a group of parasitic diseases caused by protozoan parasites from the *Leishmania* genus, which affect the human populations in tropical and subtropical regions. Different *Leishmania* species can lead to diverse clinical manifestations that depend on the host's immune competence. Both wild and domestic animals, including the domestic dog, can also be affected by leishmaniasis. The dog is considered the primary reservoir of *Leishmania infantum*, which is the aetiological agent of zoonotic visceral leishmaniasis. Although not commonly detected in dogs, *Leishmania amazonensis*, the agent of human cutaneous leishmaniasis in Central and South America, can also cause canine leishmaniasis (CanL) [1]. The dog's immune response plays a critical role in controlling parasite transmission to sandflies and limiting parasite spread within the host, influencing the disease's outcome, which can range from subclinical infection to severe systemic illness. Although it is widely recognized that an acquired immune response is crucial for controlling CanL, innate immunity, which is the first line of defense mediated by various cells, including natural killer (NK) cells and dendritic cells (DCs), is also essential.

NK cells are a granular lymphocyte subtype characterized by the loss of cluster of differentiation (CD) 3 that are differentiated in the bone marrow. Although representing only 10%–15% of blood lymphocytes, they are crucial cells of the innate response because can rapidly release immune mediators and lyse target infected cells without prior activation [2, 3]. Their activity depends on a combination of signals displayed by activating and inhibitory receptors that recognize ligands in the target cell surface. Inhibitory receptors of NK cells, as is the case of killer immunoglobulin-like receptors (KIRs) can recognize molecules of class I of the major histocompatibility complex (MHCI) [4–6]. Thus, the absence or alterations of MHCI molecules, usually found in infected cells (and in malignant cells), prevent the attachment of the inhibitory receptors, resulting in the activation of NK cells [2, 6, 7]. Once activated, NK cells can produce chemokines (e.g., macrophage inflammatory protein 1 [MIP-1], chemokine ligand 5 [CCL5]) and immune mediators (e.g., interferon [IFN]-*γ*, tumor necrosis factor [TNF]-*α*), which may favor the expansion of the type 1 T helper (Th1) immune response and macrophage activation, directing the implementation of antimicrobial mechanisms [8, 9]. Moreover, during systemic infections, NK cells can also produce interleukin (IL)-13 [10–12] in addition to IL-10, which can regulate the immune response by inhibiting IL-12 [13]. On the other hand, NK cell cytotoxic mechanisms take a lead in infection control through the exocytosis of granules rich in the soluble monomer perforin which causes pores in the membrane of the target cell. The perforin pores constitute a passive conductor for granzymes and granulysin across the cell membrane, inducing ion exchange that generates an intracellular imbalance [14, 15]. Granzyme B cleaves proteins at aspartate residues, leading to the pro-caspase-10 activation to caspase-10, which can cleave inhibited caspase-activated DNase (ICAD). Once activated, CAD transposes the nuclear membrane and can cleave DNA leading to cell death [16–18].

According to previous studies, antigen-presenting cells (APCs) can also stimulate NK cells, which can send stimulatory signals to lymphocytes [19, 20]. There are not many studies regarding the interaction between DCs and NK cells in the context of *Leishmania* infection. To date, published studies have reported that plasmacytoid DCs (pDCs) and myeloid DCs may be critical for in vivo activation of NK cells by releasing large quantities of IL-12 and IFN-*γ* [21]. Moreover, studies using the mouse model have shown that *L. major* genomic DNA and *L. braziliensis* promastigotes can induce IL-12 and IFN-*α*/*β* secretion by classical DCs and pDCs, which in turn favor the increase of IFN-*δ* and NK cell cytotoxicity [15, 22].

DCs and NK cells interact in the lymph node paracortex, where they can induce a localized CD4^+^ T cell immune response. According to Bajénoff et al. [23], NK cells from mice infected with *L. major* constitute a “network” within lymph nodes, where they interact with DCs. This interaction promotes cytokines production, which in turn shapes the development of the adaptive immune response. Although NK cell activation is usually related to disease protection [24, 25], some studies have reported that *L. amazonensis*, *L. mexicana*, and *L. tropica* amastigotes are weak stimulators of IL-12 producing DCs, explaining the limited NK cell response observed [25, 26]. On the other hand, DCs and IL-12 appear to become insufficient to activate NK cells, as the presence of IL-2-producing CD4^+^ T cells is also required [15, 27]. Interestingly, it has been shown in vivo and in vitro that the absence of specific CD4^+^ T cells after *L. major* infection prevents NK cell activation by DCs [28].

In summary, these findings stress that stimulation of immune response through DCs-NK cells crosstalk increases NK cell activation and cytotoxicity, thereby strengthening a defensive innate immune response against *Leishmania* infection. Therefore, using an in vitro dog model, the current study investigates the interplay between NK cells and *Leishmania* exposed-DCs by examining the cytokine profile and cytotoxic activity of NK cells, as well as the apoptotic state of DCs, chemokine and cytokine generation, surface expression of MHC molecules, and the gene expression costimulatory molecules. Recently, it has been recognized that extracellular vesicles (EVs) derived from *Leishmania* parasites, which carry bioactive molecules, can influence the host immune response by modulating the activity of immune cells, as is the case of DCs [29]. Thus, this study also examines the impact of EVs on DCs-NK cell interplay.

## 2. Materials and Methods

### 2.1. Experimental Design

To examine the relationship between DCs and NK cells, DCs were in vitro differentiated from blood monocytes (monocyte-derived DCs [moDCs]), and NK cells were directly isolated from the buffy coat and immunophenotyped by multiparametric flow cytometry. Then, moDCs were exposed to *Leishmania* parasites or primed with EVs shed from *Leishmania* cultured promastigotes and cocultured with autologous NK cells. The immune activity of loaded moDCs and NK cells was then analyzed. Cytokines (IFN-*γ*, TNF-*α*, and IL-13) and chemokines (CCL3/MIP-1*α*, CCL4/MIP-1*β*, and CXCL8/IL-8) gene expression quantified by real-time PCR (RT-qPCR) were used to assess NK cell activity. To analyze cytotoxicity, perforin, and granzyme B released by NK cells to the extracellular medium were evaluated by a colorimetric immunoassay, and the levels of apoptotic/necrotic moDCs were estimated by multiparametric flow cytometry. The impact of moDCs-NK cells interaction on intracellular parasite survival was examined by resuspending moDCs in Schneider's Drosophila medium to perceive parasite viability. Furthermore, the immune activity of adherent moDCs induced by active NK cells was evaluated by gene expression of proinflammatory (IL-12p40) and anti-inflammatory (IL-10) cytokines and the costimulatory molecules CD80/CD86 by RT-qPCR, in addition to the assessment of MHC surface expression by multiparametric flow cytometry, indicating possible antigen presentation to lymphocytes.

The Ethics and Welfare Committee of the Faculty of Veterinary Medicine, University of Lisbon (Portugal), approved the current study that was performed following the institutional guidelines and EU requirements. Informed consent was obtained from dog tutors who were previously notified of the work's purpose and the interventions' nature.

### 2.2. *Leishmania* Growth and EVs Purification

*L. infantum* (MCAN/2012/IHMT0003SG) and *L. amazonensis* (MHOM/BR/1973/M2269) promastigotes were grown in Schneider's Drosophila medium (SCH, Sigma–Aldrich) supplemented with fetal bovine serum heat-inactivated (hiFBS, Sigma–Aldrich) and penicillin-streptomycin (Biochrom) (complete SCHN medium). The parasites were maintained for a maximum of five passages in case of *L. infantum* and 10 passages for *L. amazonensis* to preserve virulence. Promastigotes at the late log growth phase were used to infect moDCs.

EVs derived from *L. infantum* and *L. amazonensis* were harvested according to Weber et al. [30]. Briefly, *L. amazonensis* and *L. infantum* promastigotes were grown in SCH supplemented with exosome-depleted fetal bovine serum (Gibco, USA). After 72 h of growth, cultures were centrifuged, and the supernatant free of parasites was added to the exosome isolation reagent (Invitrogen). After overnight incubation, the pellet of the centrifuged solution was resuspended in 1 × phosphate-buffered saline solution (PBS, Lonza, Belgium). The protein concentration was estimated in NanoDrop (Thermo Fisher Scientific). moDCs were exposed to promastigotes or purified EVs.

### 2.3. Differentiation of moDCs and NK Cell Isolation

**Figure 1:**
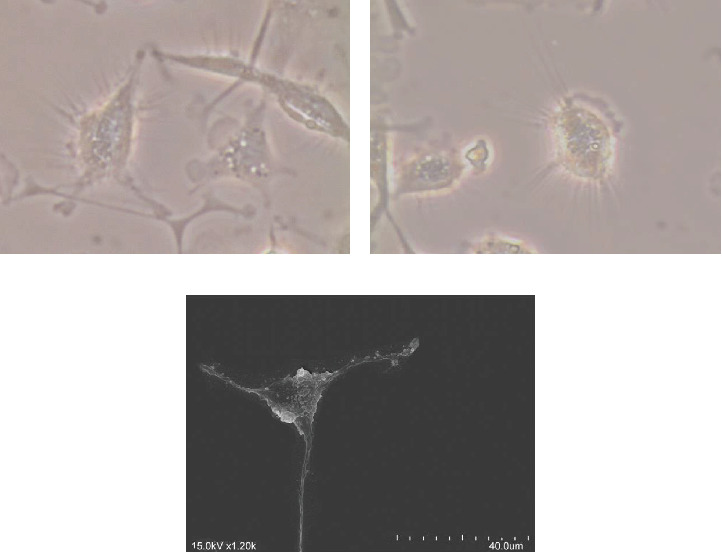
Canine monocyte-derived dendritic cells of dog. Digital images of moDCs were captured using inverted optical microscopy at 400x magnification (A, B) and scanning electron microscopy (C). moDCs, monocyte-derived DCs.

moDCs were differentiated according to the in vitro canine model described by Valério-Bolas et al. [29]. Briefly, mononuclear cells were incubated in Roswell Park Memorial Institute medium 1640 (RPMI, Sigma–Aldrich) supplemented with hiFBS, colony-stimulating factor, and recombinant canine IL-4 (rcaIL-4, R&D Systems) until showing conventional DC morphology with an elongated shape and filiform cytoplasmic emissions (Figure 1).

NK cells were isolated from the buffy coat (20–30 mL) of peripheral blood obtained from clinically and analytically healthy dogs that were also negative for *Leishmania* infection by serology and qPCR, as previously described by Valério-Bolas et al. [29].

Buffy coat was overlaid onto a Hystopaque (density 1077, Sigma–Aldrich) gradient and after centrifugation, the fraction enriched in peripheral blood mononuclear cells (PBMCs) was collected. Then, mononuclear cells were used to sort NK cells using a MultiStand System (Miltenyi Biotec, Germany) and NK Cell Isolation Kit microbeads (Miltenyi Biotec) following the manufacturer's indications. PBMCs resuspended in 1 × PBS supplemented with 0.5% hiFBS and 2 mM ethylenediaminetetraacetic acid (EDTA) (magnetic separation [MS] buffer) were added to NK Cell Biotin-Antibody Cocktail (Miltenyi Biotec) and incubated for 5 min at 4°C. MS buffer (30 μL) was added to 20 μL of NK Cell MicroBead Cocktail and incubated for 10 min at 4°C. The cell suspension was then passed through MS columns attached to a Mini MACS. Unlabeled cells, representing lymphocytes enriched in NK cells, were eluted and the column was detached from the magnetic field. This cell suspension underwent a new MS to remove CD3^+^ cells. The cells were washed (10 min, 500 × g, 4°C) and resuspended in 100 µL of PBS supplemented with 3% hiFBS and 10 mM EDTA (cell sorting buffer) and then added to MagniSort Positive Selection Antibody. Cells were incubated for 10 min at room temperature, washed with cell sorting buffer (5 min, 300 × g, 4°C), and resuspended in 100 µL of sorting buffer and 20 µL of MagniSort Positive Selection Beads. After incubation (10 min, room temperature), the cell suspension passed through the MS column attached to a Mini MACS separator, and eluted unlabeled cells were collected. Cell morphology was observed under an optic microscope, and digital images were taken.

### 2.4. Immunophenotyping and Morphology of NK Cells

Sorted NK cells immunophenotype was evaluated by multiparametric flow cytometry using five surface biomarkers (CD3, CD16, CD21, CD94, and MHCII). Cells washed twice with cold 1 × PBS (300 × g, 10 min, 4°C) were incubated (30 min, 4°C) in PBS 2% hiFBS with the following monoclonal antibodies directed conjugated: FICT-conjugated mouse antidog CD3 (clone CA17.2A12, Bio-Rad), Alexa Fluor 647-conjugated mouse anticanine CD21 (clone CA2.1D6, Bio-Rad), APC-conjugated mouse antidog CD94 monoclonal antibody (clone HP-3D9, eBioscience), FICT-conjugated Human CD16 monoclonal antibody (clone eBioCB16, eBioscience), and FICT-conjugated rat antidog DLA region MHC class II monomorphic (clone YKIX334.2, Bio-Rad) (Table S1). Cells fixed in PBS 2% paraformaldehyde (Sigma–Aldrich) were acquired on a CytoFLEX system cell analyzer (Beckman Coulter). To ensure accurate sample reading, controls were used to identify the background fluorescence, adjust channel spillover, and define the gates [31]. Debris and pyknotic cells were removed by applying the FSC-H vs. SSC-H gate, and the nonclumping cells were retired using the FSC-H vs. FSC-A singlet gate. Data were analyzed using the CytExpert software (Beckman Coulter).

### 2.5. moDCs-NK Cells' Cocultures

To establish moDCs-NK cells' cocultures, moDCs were added to 96-well plates (1 × 10^5^ cells/well) and exposed to *Leishmania* cultured promastigotes, at 3 : 1 parasite–moDCs ratio or with *Leishmania* derived EVs (10 μg/mL). After incubation for 24 h at 37°C in a humidified atmosphere containing 5% CO_2_, autologous NK cells freshly isolated were plated at a 1 : 1 moDCs-to-NK cell ratio. After 18 h of incubation, cells were pooled (500 × g, 10 min, room temperature) and the coculture supernatant was obtained to evaluate perforin and granzyme concentration by immunoassays while cells (moDCs and NK cells) were kept for RNA extraction and multiparametric flow cytometry analysis. In parallel, resting putative NK (pNK) cells, unprimed moDCs, loaded moDCs, and cocultures unloaded moDCs-NK cells were used as controls.

### 2.6. Perforin and Granzyme Immunoassays

To assess NK cell cytotoxicity, perforin, and granzyme B concentrations were estimated in the coculture supernatants using the dog/canine perforin 1 PRF1 ELISA Kit (Novatein Bioscience, USA) and Human granzyme B ELISA Kit (Quimigen Unipessoal LDA, Portugal), respectively, following the manufacturer's instructions.

### 2.7. DNA Copies of Chemokines, Cytokines, and Costimulatory Molecules

RNA isolated from NK cells cocultured with loaded moDCs (infected by *L. infantum* or *L. amazonensis* or primed by EVs) was used to examine the gene expression of chemokines (CCL4, CCL3, and CXCL8) and cytokines (IL-13, IFN-*γ*, and TNF-*α*) through RT-quantitative PCR (qPCR). On the other hand, RNA from loaded moDCs cocultured with NK cells was obtained to assess the number of copies of cytokines (IL-10 and IL-12p40) and costimulatory molecules (CD80/CD86).

NZY total RNA isolation kit (genes and enzymes) and NZY first-strand complementary DNA (cDNA) synthesis kit (genes and enzymes) were used to extract RNA and then transcribed into cDNA, respectively. DNA amplification was performed in a Bio-Rad CFX Maestro PCR System thermal cycler (Bio-Rad, UK) using endogenous *β*-actin control, as previously reported [32]. RT-qPCR conditions were 5 min at 95°C followed by 40 cycles of 30 s at 95°C, 30 s at each primer/gene-specific temperature, and 90 cycles of 10 s at 50°C with 0.5°C increase for each thermal cycle. Primers used for amplification of cDNA were either designed using Primer3 software [33] or selected from previously published studies (Table S2). External cDNA standards were used to determine the amount of DNA copies, as described by Rodrigues et al. [34].

### 2.8. Surface Expression of MHC Molecules by Loaded moDCs

Loaded moDCs in coculture with NK cells were used to analyze the MHC expression using multiparametric flow cytometry. Harvested moDCs were washed, fixed with paraformaldehyde, and incubated with the rat antidog DLA region class II (MHCII, BioRaD, YKIX334.2) and mouse antihuman HLA ABC, (MHCI, BioRad, W6/32) monoclonal antibodies conjugated with fluorescent dyes (Table S1). Cells were then rewashed and acquired on a flow cytometer, and the mean fluorescence intensity (MFI) of labeled cells was assessed.

### 2.9. Apoptosis of moDCs

The cytotoxic effect of NK cells on moDCs was assessed using annexin and propidium iodide (PI) to label the cells. Loaded moDCs were incubated for 24 h before adding to NK cells. After 18 h of incubation, moDCs were washed with cold 1 × PBS (300 × g, 10 min, 4°C), incubated with annexin (TACSTM Annexin V FITC, R&D Systems, USA) following manufactured instructions, and then treated by PI (R&D Systems). The proportion of apoptotic cells (FITC^+^ or ^−^ /PI^+^ cells) was determined by multiparametric flow cytometry (Table S3).

### 2.10. Viability of Intracellular Parasites

The viability of *L. amazonensis* and *L. infantum* parasites taken up by moDCs was assessed after they had been in contact with the NK cells. After 24 h of infection, moDCs were cocultured with NK cells for 18 h. The harvested moDCs were washed twice with 1 × PBS to remove any extracellular parasites and incubated in the complete SCH medium for 72 h at 24°C to allow the differentiation of intracellular viable amastigotes into extracellular motile promastigotes. The number of viable parasites was estimated using a Neubauer counting chamber under an optical microscope.

### 2.11. Statistical Analysis

A total of six canine samples were collected and each sample was analyzed in duplicate. Data statistical analysis was performed by applying the nonparametric Wilcoxon test for paired samples using GraphPad Prism software (USA). Significant statistical differences were considered whenever the *p*-value was less than 0.05 (5% significance level).

## 3. Results

### 3.1. Magnetically Sorted Cells Were Enriched in pNK Cells

**Figure 2:**
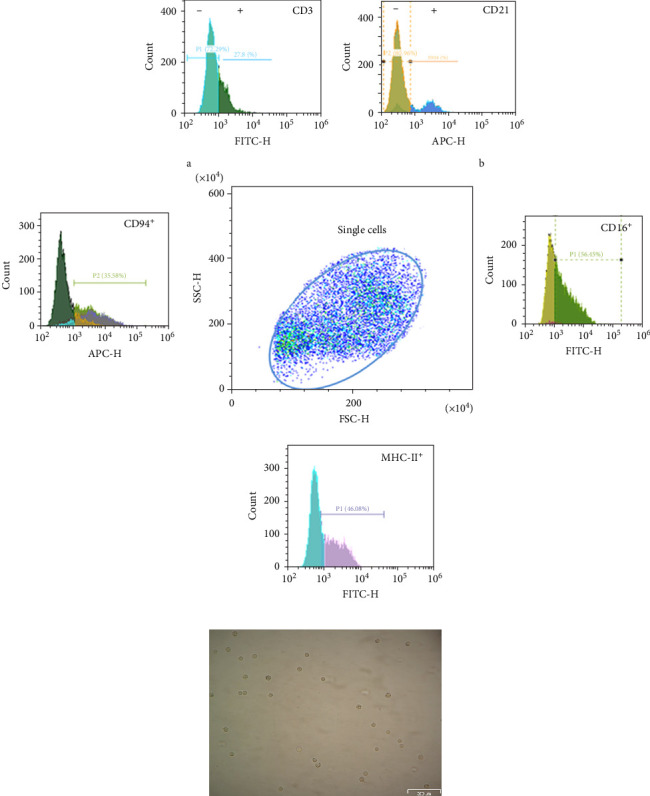
Morphology and immunophenotype of NK cells. Negative magnetic selection was used to isolate NK cells from dog PBMCs. NK cells were labeled with CD3, CD16, CD21, CD94, and MHCII mononuclear antibodies directed conjugated and analyzed using multiparametric flow cytometry (A). Representative single-cell dot plots and histograms of labeled cell populations are shown. A digital image of sorted NK cells observed by inverted light microscopy is included (B).

The cell population sorted from PBMCs was immunophenotyped using the surface markers CD3, CD21, CD16, CD94, and MHCII (Figure 2A). Although the molecular signature of canine NK cells remains controversial, CD16, CD94, and MHCII molecules are expected to be present on the cell surface, but not CD21. Nonetheless, it has also been indicated that depending on maturation state CD3 expression can also occur in NK cells [35]. Thus, in the current study, CD3, CD16, CD94, and MHCII were used as molecular markers of pNK cells of dogs. Within the cell population magnetically sorted, there was ≈27% of CD3^+^CD21^−^CD16^+^CD94^+^MHCII^+^ corresponding to immature NK cells and ≈54% of CD3^−^CD21^−^CD16^+^CD94^+^MHCII^+^ cells which represent the most consensual phenotype for mature NK cells, indicate that it was obtained a cell population enriched in pNK cells. These cells appeared morphologically homogenous under light microscopy, showing a round shape and small to medium sized. They were free of contamination from larger mononuclear cells or granulocytes (Figure 2B).

### 3.2. moDCs Infected With *L. infantum* or *L. amazonensis* Drive the Generation of Chemokines by pNK Cells

NK cells can produce chemokines either by interacting with other immune cells or in direct response to pathogens. To analyze the capacity of pNK cells to attract other leukocytes following coculture with moDCs infected by *Leishmania* parasites or primed with parasite-derived EVs, the gene expression levels of CCL3, CCL4, and CXCL8 (IL-8) were assessed using RT-qPCR.

**Figure 3:**
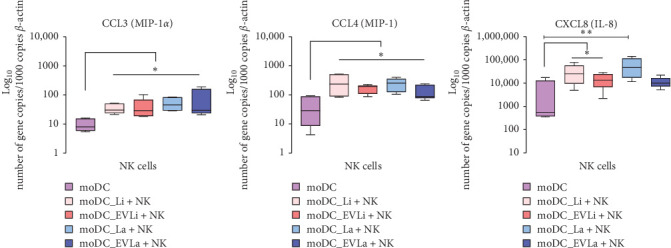
Chemokine gene expression in pNK cells induced by loaded moDCs. CCL3 (A), CCL4 (B), and CXCL8 (C) gene expression in pNK cells were evaluated by RT-qPCR following stimulation by moDCs infected with *L. infantum* (moDC+Li) or *L. amazonensis* (moDC+La) parasites, or moDCs primed by EVs (moDC+LaEVs or moDC+ LiEVs). Gene expression of pNK chemokines induced by unloaded moDCs was also assessed (moDC). Data from six samples (*n* = 6) with two replicates per sample are shown as box plots (interquartile range, median, maximum, and minimum values) with statistically significant differences indicated by *⁣*^*∗*^*p* < 0.05 and *⁣*^*∗∗*^*p* < 0.01. moDCs, monocyte-derived DCs.

Results indicated that moDCs primed with EVs induced significant upregulation of *CCL3* (*p*=0.0313) and *CCL4* (*p*=0.015) in pNK cells (Figure 3A,B) when compared to pNK that were in coculture with unloaded moDCs. Likewise, infected moDCs induced marked overexpression of *CCL3* (*p*=0.031) and *CCL4* (*p*=0.0156) in pNK cells. Although CXCL8 mRNA levels increased in pNK cells (Figure 3C), significant upregulation was observed in pNK cells cocultured with moDCs infected with *L. infantum* (*p*=0.0391) or *L. amazonensis* (*p*=0.0078) and in moDCs primed with *L.infantum* derived EVs (*p*=0.0363).

Overall, the results suggest that loaded moDCs can direct pNK cells to generate chemokines, mainly CCL4 and CCL3, which attract CD4^+^ and CD8^+^ T cells, respectively. Furthermore, pNK triggered by infected moDCs or *L. infantum* derived EVs can generate CXCL8, which is chemotactic for polymorphonuclear cells.

### 3.3. *L. amazonensis* Infected moDCs Induce pNK Cells to Generate IFN-*γ* and IL-13

Activated NK cells can release cytokines in response to various stimuli, influencing the immune response of lymphocytes. Thus, to examine the proinflammatory activity of pNK cells, RT-qPCR was used to quantify the gene expression of IL-13, IFN-*γ*, and TNF-*α* in pNK cells cocultured with *Leishmania*-infected moDCs and EVs primed moDCs.

**Figure 4:**
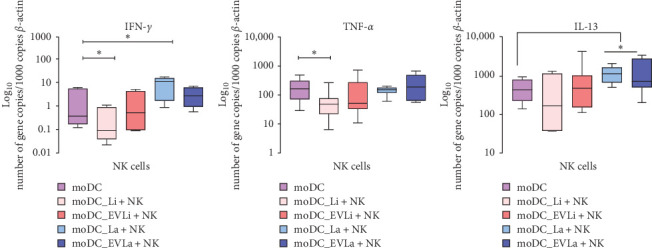
Cytokine gene expression in pNK cells cocultured with loaded moDCs. Gene expression of IFN-*γ* (A), TNF-*α* (B), and IL-13 (C) in pNK cells induced by moDCs infected with *L. infantum* (moDC+Li) or *L. amazonensis* (moDC+La) parasites or primed with EVs (moDC+LiEV or moDC+LaEV) was evaluated using RT-qPCR. Cytokine gene expression in pNK cells cocultured with unprimed moDCs (moDC) was also assessed. Data from six dog samples (*n* = 6) with two replicates per sample are presented as box plots showing the interquartile range, median, maximum, and minimum values. Statistically significant differences are indicated by *⁣*^*∗*^*p* < 0.05. moDCs, monocyte-derived DCs.

In comparison to pNK cells cocultured with unloaded moDCs, *IFN-γ* was significantly upregulated (*p*=0.0313) in pNK cells induced by moDCs infected with *L. amazonensis* (Figure 4A). In turn, coculture with moDCs exposed to *L. infantum* parasites led to a marked downregulation of *TNF-α* (*p*=0.0273) and *IFN-γ* (*p*=0.0156) in pNK cells (Figure 4A,B). Additionally, a significant accumulation of IL-13 mRNA was only found in pNK cells induced by moDCs infected with *L. amazonensis* parasites (*p*=0.015) or primed with *L. amazonensis* derived EVs (*p*=0.0313) (Figure 4C).

Therefore, these results suggest that *L. infantum* infected moDCs drive pNK cells to download proinflammatory cytokines. By contrast, *L. amazonensis* infected moDCs direct pNK cells to overexpress IFN-*γ* and IL-13, without influencing TNF-*α* gene expression, while moDCs primed with *L. amazonensis* derived EVs drive pNK cells to generate IL-13. However, moDCs primed by *L. infantum* EVs have no impact on cytokine generation by pNK cells.

### 3.4. NK Cell Degranulation Is Triggered by *L. amazonensis* and EVs Loaded moDCs

NK cell cytotoxicity is an effector mechanism of the innate immune response that can damage pathogens and infected cells throughout the exocytosis of granules containing perforin and granzymes. Therefore, to evaluate the cytotoxic activity of pNK cells, the levels of perforin and granzyme B released into coculture supernatants were estimated using colorimetric immune assays.

**Figure 5:**
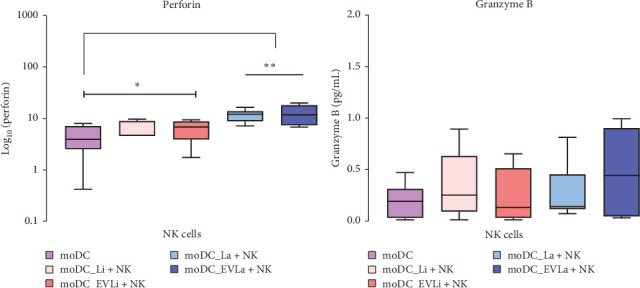
Degranulation of pNK cells induced by loaded moDCs. The release of perforin (A) and granzyme B (B) by pNK cells stimulated by moDCs infected with *L. infantum* (moDC+Li) or *L. amazonensis* (moDC+La) parasites, or primed by EVs (moDC+LaEVs or moDC+LiEVs) were evaluated using colorimetric immunoassays. Perforin and granzyme B released by pNK cells induced by unloaded moDCs (moDC) were also assessed. Data from six canine samples (*n* = 6) with two replicates per sample are represented by box plots showing interquartile range, median, maximum, and minimum values. Statistically significant differences are indicated by *⁣*^*∗*^*p* < 0.05 and *⁣*^*∗∗*^*p* < 0.01. EVs, extracellular vesicles; moDCs, monocyte-derived DCs.

Compared to pNK cells cocultured with unloaded moDCs, increased levels of perforin were found in all moDCs cocultures (Figure 5A). Indeed, the amount of perforin in supernatants of pNK cells cocultured with *L. amazonensis* (*p*=0.0078) or EVs loaded moDCs was significantly high (*p*_LiEVs_ = 0.0156, *p*_LaEVs_ = 0.0078). In contrast, no significant differences were observed in granzyme B levels in all loaded moDCs cocultures compared to unloaded moDCs cocultures (Figure 5B).

Thus, *L. amazonensis* or EVs-loaded moDCs induce pNK cell degranulation, mainly resulting in the release of perforin. However, the process of pNK degranulation triggered by moDCs exposed to *L. infantum* appears to be more regulated, leading to lower levels of perforin. The early release of perforin into the extracellular environment by pNK cells is complemented by a minimal release of granzyme B.

### 3.5. Infected moDCs and Intracellular Parasites Are Resistant to pNK Cell Cytotoxicity

Considering that NK perforin can promote pore formation in the membranes of target cells, resulting in loss of membrane integrity and leading to osmotic imbalance, the cytotoxic effect of pNK cells in loaded moDCs was assessed by estimating the proportion of apoptotic cells. The results were normalized to the frequency of loaded apoptotic moDCs that had not interacted with NK cells, thereby minimizing the direct impact of parasites or EVs on moDCs apoptosis. Moreover, the viability of *L. amazonensis* and *L. infantum* parasites internalized by moDCs was also examined by estimating extracellular motile promastigote forms and assessing parasite replication.

**Figure 6:**
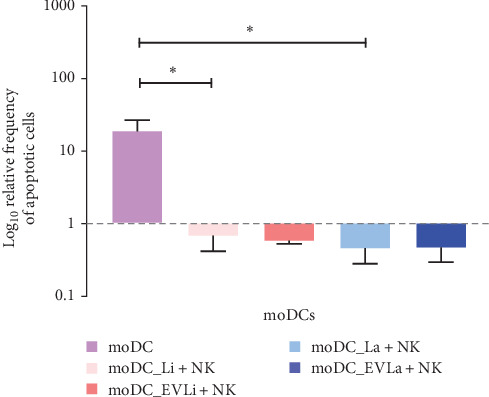
Cytotoxicity of pNK cells on loaded moDCs. Apoptotic levels of moDCs infected with *L. infantum* (moDC+Li) or *L. amazonensis* (moDC+La) parasites, or primed with EVs (moDC+LaEVs or moDC+LiEVs) that were cocultured with pNK cells were assessed using multiparametric flow cytometry after staining with annexin-V and PI. In parallel, the cytotoxic effect of pNK cells on unloaded moDCs (moDC) was also evaluated. The mean and SEM of the relative frequency of apoptotic moDCs derived from six dog samples (*n* = 6) with two replicates per sample normalized to loaded or unloaded moDCs not exposed to NK cells are represented by column bars. Statistically significant differences are indicated by *⁣*^*∗*^*p*  < 0.05. EVs, extracellular vesicles; moDCs, monocyte-derived DCs; PI, propidium iodide.

In comparison to unloaded moDCs cocultured with autologous pNK cells, the levels of apoptotic cells were lower among cocultures involving infected or EVs primed moDCs. However, a significant reduction in cells was observed among moDCs infected with *L. infantum* (*p*=0.0150) or *L. amazonensis* (*p*=0.0156) (Figure 6).

**Figure 7:**
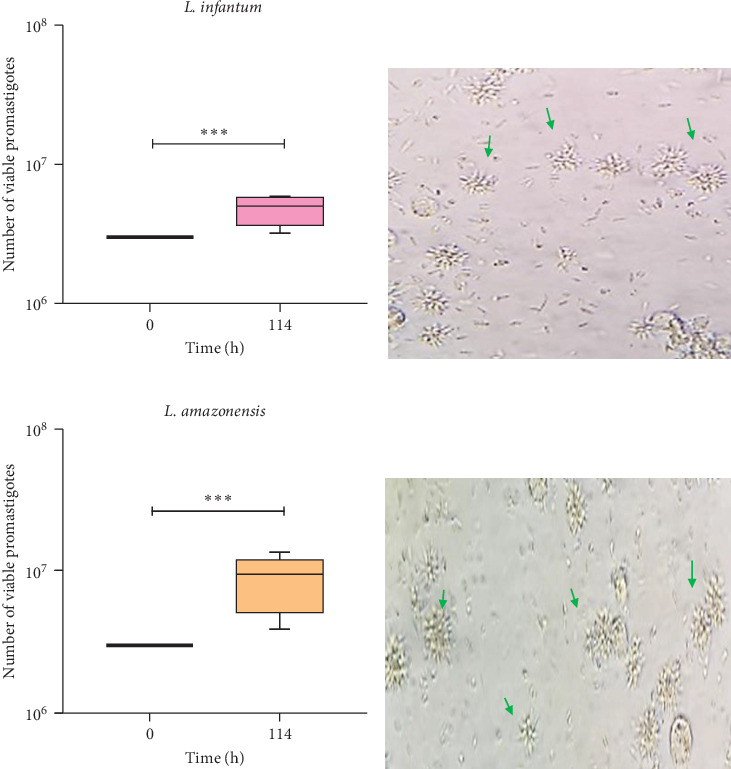
Viability of intracellular parasites in moDCs coculture with autologous pNK cells. moDCs infected with *L. infantum* or *L. amazonensis* promastigotes for 24 h were incubated with pNK cells for an additional 18 h. The cultures were then transferred to SCH and incubated for 72 h. The number of viable promastigotes (A, C) of six dog samples (*n* = 6) with two replicates per sample is presented as box plots showing interquartile range, median, maximum, and minimum values. Statistically significant differences between 0 h (initial concentration of *Leishmania* promastigotes used to infect moDCs) and 72 h (114 h total) are indicated with *⁣*^*∗∗∗*^*p* < 0.001. Digital light microscopy images of viable *L. infantum* (B) and *L. amazonensis* (D) promastigotes (400x magnification) were captured. Arrows indicate replicating promastigotes (rosettes). moDCs, monocyte-derived DCs.

Intracellular amastigotes within moDCs were able to differentiate into motile promastigotes, resulting in a significantly higher number of viable parasites (*p*_Li_ = 0.002; *p*_La_ = 0.001) compared to the initial concentration of promastigotes used to infect moDCs (Figure 7A,C). Moreover, the parasites maintained their ability to replicate (*p*_Li_ = 0.0020; *p*_La_ = 0.0010), as indicated by the high density of rosettes observed (Figure 7B,D).

Thus, infected moDCs and internalized *L. infantum* and *L. amazonensis* parasites are resistant to the cytotoxic effect of pNK cells.

### 3.6. Exposure to pNK Cells Does Not Favor Antigen Presentation by Loaded moDCs

Given the importance of the interplay between NK cells and DCs in shaping the adaptive immune response, the influence of pNK cells on antigen presentation by loaded moDCs was examined by evaluating the expression levels of MHCI and MHCII molecules along with the gene expression of CD80 and CD86 in moDCs. The results were then normalized against the levels of MHCI and MHCII surface expression as well as CD80 and CD86 gene expression in loaded moDCs (infected or EVs primed) that were not exposed to pNK cells to withdraw the direct effects of parasites or EVs.

**Figure 8:**
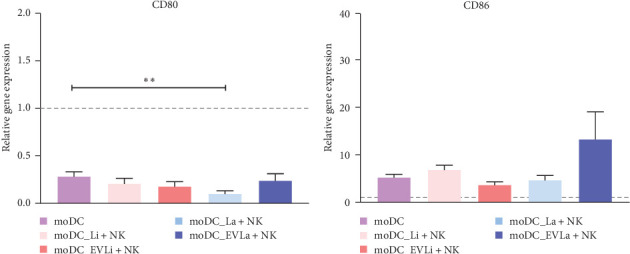
Gene expression of costimulatory molecules in loaded moDCs induced by pNK cells. Copy numbers of CD80 (A) and CD86 (B) in moDCs infected with *L. infantum* (moDC+Li) or *L. amazonensis* (moDC+La) parasites or primed with EVs (moDC+LiEVs or moDC+LaEVs) were evaluated by RT-qPCR following coculture with pNK cells. Gene expression of unloaded moDCs in coculture with pNK cells was also assessed (moDC). Column bars represent the mean and SEM of relative gene expression of six samples (*n* = 6) with two replicates per sample normalized to loaded or unloaded moDCs not exposed to NK cells. Statistically significant difference is indicated with *⁣*^*∗∗*^*p* < 0.01. moDCs, monocyte-derived DCs.

When in coculture with pNK cells, the expression levels of MHCI and MHCII molecules in loaded moDCs, along with CD80 gene expression (Figure 8A) showed no considerable changes, except for a significant reduction in CD80 gene expression (*p*=0.002) in moDCs infected with *L. amazonensis*. The accumulation of CD86 mRNA in moDCs infected with *L. amazonensis* or *L. infantum* parasites or primed by *L. infantum* derived EVs was similar to that of unloaded moDCs cocultured with pNK cells. Although the difference was not statistically significant, moDCs primed with *L. amazonensis* derived EVs showed a marked increase in CD86 gene expression (Figure 8B) compared to unloaded moDCs cocultured with autologous pNK cells.

While pNK cells do not trigger the expression of MHC and costimulatory molecules in infected moDCs or moDCs primed by *L. infantum* derived EVs, there appears to be a trend for pNK cells to favor CD86 overexpression in moDCs primed by *L. amazonensis* EVs.

### 3.7. Exposure to Autologous NK Cells Enhances the Upregulation of *IL-10* in *L. infantum* Infected moDCs and moDCs Primed by *L. amazonensis* Derived EVs

Since cytokines released by NK cells can modulate DC activity, the effect of autologous pNK cells on pro- and anti-inflammatory cytokines generated by loaded moDCs was assessed by RT-qPCR. The results were normalized to the levels of cytokine gene expression in infected and EV primed moDCs that were not exposed to NK cells.

**Figure 9:**
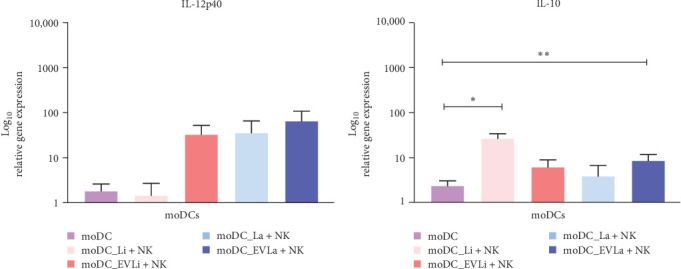
Cytokine gene expression in loaded moDCs cocultured with autologous pNK cells. IL-12p40 (A) and IL-10 (B) gene expression in moDCs infected with *L. infantum* (moDC+Li) or *L. amazonensis* (moDC+La) parasites or primed by EVs (moDC+LaEVs or moDC+LiEVs) were evaluated by RT-qPCR. Unloaded moDCs cocultured with pNK cells were also assessed. The relative gene expression of six dog samples (*n* = 6) with two replicates per sample normalized to loaded or unloaded moDCs not exposed to NK cells is presented by column bars showing mean and SEM. Statistically significant differences are indicated with *⁣*^*∗*^*p*  < 0.05 and *⁣*^*∗∗*^*p* < 0.01. EVs, extracellular vesicles; moDCs, monocyte-derived DCs.

Loaded moDCs exposed to pNK cells showed an upregulation of *IL-12p40* (Figure 9A) and *IL-10* (Figure 9B). While, IL-12p40 gene expression was similar in both *L. infantum* infected moDCs and unprimed moDCs, *L. amazonensis* infected moDCs and EVs primed moDCs cocultured with autologous pNK cells showed an accentuated IL-12p40 upregulation. Though, these increases were not statistically significant in comparison to unloaded moDCs cocultured with pNK cells. Furthermore, moDCS infected with *L. infantum* (*p*=0.0273) or primed with *L. amazonensis* derived EVs (*p*=0.0098) showed a marked upregulation of *IL-10* compared to unloaded moDCs cocultured with autologous pNK cells. Interestingly, moDCs infected with *L. infantum* parasites exhibited the highest upregulation of *IL-10* and the lowest *IL-12p40* copy number.

Thus, these results suggest that pNK cells can influence loaded moDCs to generate cytokines, particularly when moDCs are primed by EVs. Curiously, *L. infantum* infected moDCs mainly generate the regulatory cytokine IL-10, while *L. amazonensis* infected moDCs predominantly upregulate IL-12p40.

## 4. Discussion

As APCs, DCs bridge the innate and acquired immune responses, leading to the development of a T lymphocyte immune response. Furthermore, NK cells, which are a primary source of IFN-*γ*, have been shown to play a protective role in leishmaniasis by promoting a Th1 immune response [26, 28]. However, in diffuse cutaneous leishmaniasis, there was a reduction of NK cells and low levels of proinflammatory immune mediators, such as IFN-*γ* and TNF-*α*, when compared to localized cutaneous leishmaniasis [26]. On the other hand, NK cells may also play a role in the immunopathology of leishmaniasis, mainly due to the release of granzyme B, which enhances the cytotoxicity found in cutaneous leishmaniasis patients [36].

Exposure to pathogens or stimulation by cytokines and accessory cells can induce NK cells to secrete cytokines. Previous studies have shown that pathogens can directly activate NK cells either by binding the NK cell receptors or by signaling the downstream pathways of pattern recognition receptors [37, 38]. However, DCs appear to be the most effective stimulator, triggering a key role in the in vivo activation of NK cells [39–41].

The bidirectional interplay between DCs and NK cells can promote the maturation and activation of both cell types, as well as cytokine production. Previous in vivo studies using mouse models of visceral and cutaneous leishmaniasis have reported that, during the early phase of infection, DCs can activate NK cells in a TLR9 and IL-12-dependent manner, thereby limiting parasite spread and preventing disease establishment [15, 23]. Activated NK cells can release chemokines (e.g., CCL3, CCL4, CCL5) and cytokines (e.g., GM-CSF, IFN-*γ*, TNF-*α*) [40]. However, activation of DCs by NK cells requires cell-to-cell contact along with the presence of both TNF-*α* and IFN-*γ*. According to Ferlazzo et al. [39], under conditions of reduced inflammation, NK cells may be essential in supporting the effective activation of DCs, which favor a T cell immune response, but only when NK cell activation is mediated by target cell recognition. Furthermore, IL-12-producing DCs are also crucial for NK cell activation and IFN-*γ* synthesis while TNF and IFN-*γ* releasing NK cells promote the development of a Th1 immune response by enhancing DCs' costimulatory molecules [41].

The control of all clinical forms of leishmaniasis relies on T cell differentiation driven by IL-12 and the recruitment of IFN-*γ*-producing Th cells to the site of infection [42]. Helper T cells are also essential for triggering macrophages to kill intracellular amastigote forms [43] in addition to signaling pathways that are involved in reducing hyperinflammation and supporting tissue repair [44]. Moreover, *Leishmania* parasites use sophisticated strategies to manipulate the activity of macrophages and DCs in order to guarantee their own survival and replication. The primary strategy of *Leishmania* parasites involves suppression of IL-12 production, which prevents Th1 differentiation and avoids parasite antigen presentation by DCs to T cells, leading to a weakened cell-mediated immune response. The findings of the present work indicate that *L. amazonensis* infected moDCs stimulate canine pNK cells to generate IFN-*γ*. Although these infected moDCs drive pNK cells to slightly upregulate *IL-12p40*, which is an IL-12 subunit that is known to be a chemoattractant for macrophages and activated DCs [45]. Indeed, a previous study by Xin, Li, and Soong [26] reported that the upregulation of *IL-12p40* in moDCs infected with *L. amazonensis* was rather low and transient. Nevertheless, IFN-*γ* may help to restrain *L. amazonensis* infection in dogs. In mouse models of visceral leishmaniasis, as well as in dogs and humans, TNF-*α* appears to play a critical role in the onset of granuloma formation, which is associated with the control of *L. infantum* infection [46–48]. However, in the current study, the interaction between loaded moDCs with pNK cells did not lead to a considerable generation of TNF-*α*. Therefore, since the activation of inflammatory macrophages requires IFN-*γ* and TNF-*α* synergy, optimal activation of these cells should not be achieved, which may allow parasite survival. Nevertheless, NK cells seem to have divergent protagonism according to leishmaniasis clinical forms and disease severity. It has been reported that patients with diffuse cutaneous leishmaniasis exhibited a reduced number of NK cells, lower levels of cytokines, such as IFN-*γ*, and diminished TLR expression, as is the case of TLR1, TLR2, and TLR6. In contrast, patients with localized cutaneous leishmaniasis have higher cytokine levels and a greater amount of NK cells [49, 50]. Furthermore, CD4^+^ T cell-deficient mice indicate that IFN-*γ* production by NK cells is not sufficient to restrain *L*. *major* infection [51].

IL-13 is an anti-inflammatory cytokine linked to susceptibility to leishmaniasis caused by different species of *Leishmania*. Indeed, studies involving IL-13 knockout and transgenic mice have shown that this cytokine plays a key role in inducing the differentiation of Th2 cells [52]. Since IL-13 regulates inflammatory macrophages, the early production of this cytokine during *L. amazonensis* infection might be part of the strategy used by the parasite to evade host immune defenses. Interestingly, despite the early anti-inflammatory response, still appears to be able to generate IFN-*γ*. These data correlate with *L. braziliensis* infection in both mice and humans, where predominate a Th1 response after a brief period of impaired immune defenses [53]. The study of disease outcomes in IL-13 knockout and wild-type C57BL/6 mice revealed an IL-13-independent role in *L. mexicana* infection. After 8 weeks of infection, lesions that were comparable in size to those of wild-type animals were found in IL-13 knockout mice. However, while wild-type mice exhibited progressive lesion development, knockout mice healed the lesions favored by the establishment of a Th1 response. It is therefore possible that IL-4 plays a decisive role in the initial stages of lesion development, while IL-13 promotes a chronic, nonhealing form of the infection [54]. Recent findings indicate that IL-13 is a dominant Th2 cytokine in human lesions caused by *L. guyanensis*, inhibiting the expression of the *β*2 chain of the IL-12 receptor in lymphocytes [55]. In another study, Zaatar, Simaan, and Karam [56] showed that *L. major* infected mice treated with IL-13 display an increase in parasite burden. Moreover, 12 days after IL-13 administration, IFN-*γ* levels were significantly reduced, suggesting that exogenous IL-13 hinders a shift from Th1 to Th2 by preventing IFN-*γ* production, a process linked to disease severity. Coêlho et al. [57] demonstrated that human PBMCs release high levels of IL-13 and IL-10 alongside reduced IL-12 production, several hours after exposure to *L. amazonensis* parasites, promoting the activation of a Th2 response. In the present study, moDCs infected with *L. amazonensis* parasites or primed by *L. amazonensis* derived EVs induce pNK cells to generate IL-13, pointing toward an anti-inflammatory response that favors skin remodeling through fibroblast recruitment and enhancing collagen deposition. However, pNK cells also appear to be able to generate IFN-*γ*, which induces a Th1 response. Therefore, during *L. amazonensis* infection in dogs, the interplay established between DCs and NK cells may shape a balanced proinflammatory and anti-inflammatory immune response, preventing inflammation and favoring the establishment of a chronic infection.

Another immune mediator that can limit NK cell activity after *Leishmania* infection is IL-10. This immunosuppressive cytokine can be secreted by macrophages, as well as by helper and regulatory T cells [58–61], in addition to human and mouse-activated NK cells, including those from patients diagnosed with localized cutaneous leishmaniasis [62, 63]. Nevertheless, the role played by IL-10 in either activating or suppressing NK cell activity in leishmaniasis remains undefined. Indeed, during *Leishmania* infection, macrophages can antagonize NK cell functions by producing IL-10, which suppresses activating cytokines. In the current study, loaded moDCs induced by pNK cells present increased levels of IL-10 mRNA, particularly in *L. infantum* infected moDCs, which suggests the impairment of pNK activity, especially regarding the secretion of proinflammatory cytokines. Indeed, pNK cells exposed to *L. infantum* infected moDCs exhibited suppressed generation of the proinflammatory cytokines IFN-*γ* and TNF-*α*.

Both CCL3 and CCL4, which can be released by human NK cells, stimulate T cells through CCR5, inducing lymphocyte migration. CCL3 preferentially mediates recruitment of CD8^+^ T lymphocytes while CCL4 is involved in the migration of CD4^+^ T cells [64–66]. Prior research demonstrated that bone marrow-derived DCs from mice infected with *L. amazonensis* amastigotes can secrete CCL3, CCL5, and CXCL10 when in coculture with NK cells [67, 68]. In turn, the current study findings suggest that moDCs infected with *L. amazonensis* or *L. infantum* parasites or primed by EVs direct the generation of both CCL3 and CCL4 by pNK cells. These chemokines are likely to promote the recruitment of lymphocytes to the site of infection, thereby favoring a cellular immune response (helper and cytotoxic) in the parasite target organs, which may limit the infection in dogs. The production of the chemokine CXCL8 by both leukocytes and nonleukocyte somatic cells appears to rely on IL-1 proinflammatory stimuli. This chemokine plays a crucial role in inducing the migration of polymorphic mononuclear cells [69, 70], which are key players involved in the first line of defense against *Leishmania* infection. In the current work, infected moDCs and *L. infantum* EVs primed moDCs stimulate NK cells to upregulate CXCL8, suggesting the potential recruitment of polymorphic mononuclear cells to parasite target organs, which may elicit parasite load reduction. An in vitro study reported by Pereira et al. [71] highlights the effector functions of canine polymorphic mononuclear cells during the early phase of *L. infantum* infection, promoting parasite load reduction through the emission of extracellular traps.

The interaction between DCs and NK cells can drive the differentiation and proliferation of T cells [72, 73]. The maturation of DCs is associated with the overexpression of surface markers, such as MHCII and costimulatory molecules (e.g., CD80, CD86). These molecules induce T cell receptor signaling, enabling T helper cells to recognize antigens complex with MHC molecules [74]. According to Calmeiro et al. [75], immature DCs cocultured with NK cells and lipopolysaccharide lead to the maturation of DCs, overexpressing CD86 and releasing IL-12. NK cells recognize target cells through surface receptors that interact with MHCI epitopes. Recognition of these epitopes prevents NK cell cytotoxicity, but if MHCI molecules on the target cell differ from those of the other cells of the organism, NK cell cytotoxicity is triggered. During moDCs maturation, overexpression of MHCI may protect these cells from NK lysis [76, 77]. In the current study, pNK cells do not influence the expression of MHC surface molecules of loaded moDCs and do not appear to affect the gene expression of costimulatory molecules, beyond the modulation exerted by parasites or EVs. Except in the case of moDCs infected with *L. amazonensis*, where pNK cells lead to diminished CD80 gene expression. Thus, pNK cells do not influence loaded moDCs to trigger the activation of the T cell immune response.

In addition to producing immune mediators following activation by DCs, a key activity of NK cells is to promote the destruction of infected cells by the exocytosis of granules, which release perforin and granzyme, leading to the apoptosis of target cells. In *Leishmania* infection, granzymes play a role in enhancing inflammation, which contributes to tissue damage [78]. Therefore, the effect of the cytotoxic activity of pNK cells on moDCs and the viability of parasites internalized by moDCs was also examined in the present study. Although perforin and granzyme B were released by pNK cells when induced by loaded moDCs, reduced levels of apoptotic cells were exhibited by infected moDCs. Furthermore, internalized parasites were viable and maintained their replicative capacity, being able to establish infection. Thus, the cytotoxic activity of canine NK cells can be impaired by *L. infantum* and *L. amazonensis* infected moDCs.

## 5. Conclusions

**Figure 10:**
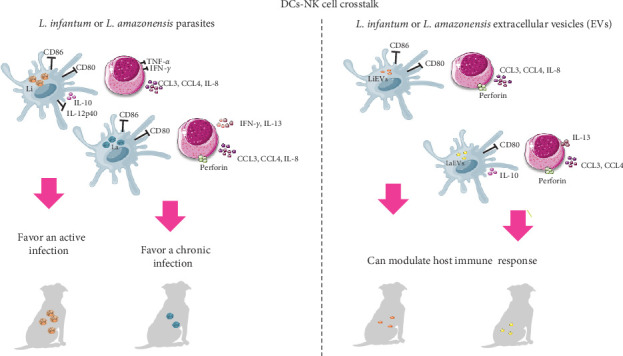
Proposed model illustrating the in vitro crosstalk between DCs and NK cells during *Leishmania* infection. DCs infected with *L. infantum* (Li) or *L. amazonensis* (La), or primed with EVs derived from *L. infantum* (LiEVs) or *L. amazonensis* (LaEVs) activate innate immune cells through distinct pathways. Loaded DCs induce NK cells to generate chemokines, which can attract other immune cells to the site of infection. The interplay between *L. infantum* infected moDCs and NK cells promotes the generation of the immunosuppressive cytokine IL-10, which can favor active infection. In contrast, *L. amazonensis* drives a mix of anti- and pro-inflammatory cytokines associated with perforin release, promoting a balanced immune response, that may limit infection and favor the establishment of chronic disease. DCs primed with parasite derived EVs stimulate the upregulation of anti-inflammatory cytokines, highlighting the role of EVs in shaping the early immune response. EVs, extracellular vesicles; moDCs, monocyte-derived DCs.

In CanL, the intricate interactions that are established between the dog's immune response and parasite control have yet to be fully understood. NK cells can establish multiple interactions with innate leukocytes including DCs, driving the killing of target cells. Given the scarce information available on the interplay between DCs and NK cells in leishmaniasis, this study reports for the first time the immune interaction features of canine DCs and NK cells during infection by cutaneous and visceral *Leishmania* parasites, as well as by parasite-derived EVs. An in vitro model was established using moDCs infected with *L. infantum*, which is the *Leishmania* species that is the primary cause of CanL worldwide, or infected with *L. amazonensis*, a species that has also been found in dogs. These moDCs were then exposed to autologous pNK cells to study their interactions. In addition, moDCs primed with EVs derived from both parasite species were also used. Chemotaxis, cytotoxicity, immune mediators, MHC molecules, and costimulatory molecules that are involved in T cell activation and infection control have been studied. The findings suggest that the interplay between canine moDCs and pNK cells, although exhibiting different immune response patterns, appears to be regulated by the cutaneous and visceral *Leishmania* spp., favoring the establishment of active or chronic infections (Figure 10). Crosstalk of *L. amazonensis* infected moDCs and pNK cells leads to a balanced pro- and anti-inflammatory response involving perforin release, which may control lesion outcome, as lesion healing relies on parasite control and modulation of local inflammation. While *L. infantum* infected moDCs contribute to a regulatory environment that supports parasite survival. *Leishmania* EVs, which are intrinsic to the parasite's biology, may be used to manipulate infection outcomes by modulating the interplay between DCs and NK cells. EVs induce a DCs-NK cell crosstalk similar to the respective parasites, but differ from parasite infection in a few aspects. *L. infantum* EVs do not favor cytokine generation while *L. amazonensis* EVs can foster an immunosuppressive environment. Moreover, EVs derived from both parasite species are chemotactic, having the potential to attract other leukocytes to the infection site. Thus, EVs emitted by *Leishmania* parasites are emerging as key immunomodulators of the canine immune response.

## Data Availability

The data that support the findings of this study are available upon request from the corresponding author. The data are not publicly available due to privacy or ethical restrictions.

## Supporting Information

Supporting Information The panel of direct conjugated monoclonal antibodies used to immunophenotype pNK cells and DCs by multiparametric flow cytometry is listed in Table S1, including the laser and emission filters used. The reverse and forward primers used in RT-qPCR to assess gene expression of chemokines (CCL4, CCL3, and CXCL8), cytokines (IFN-, TNF-*α*, IL-3, IL-10, IL-12p40, and IL-13), and costimulatory molecules (CD80 and CD86) in moDCs and pNK cells are listed in Table S2. The primers for amplifying the housekeeping gene (*β*-actin) are also included. Table S3 includes a list of reagents used in the current study, the vendors, catalog number, and concentration.
