# Supplementary material for: Unveiling the Interplay Between Dendritic Cells and Natural Killer Cells as Key Players in Leishmania Infection
Source: J Immunol Res. 2025 Feb 10;2025:3176927. doi: 10.1155/jimr/3176927 (PMC11832263; doi:10.1155/jimr/3176927)
Supplement: Supporting Information — The panel of direct conjugated monoclonal antibodies used to immunophenotype pNK cells and DCs by multiparametric flow cytometry is listed in Table S1, including the laser and emission filters used. The reverse and forward primers used in RT-qPCR to assess gene expression of chemokines (CCL4, CCL3, and CXCL8), cytokines (IFN-, TNF-α, IL-3, IL-10, IL-12p40, and IL-13), and costimulatory molecules (CD80 and CD86) in moDCs and pNK cells are listed in Table S2. The primers for amplifying the housekeeping gene (β-actin) are also included. Table S3 includes a list of reagents used in the current study, the vendors, catalog number, and concentration. [file 3176927.f1.pdf]

Supplementary Materials

Laser	488 nm				640 nm	
**Emission filters**	525/40	525/40	525/40	585/42	660/20	665/20
**Flurochrome**	FITC	FITC	FITC	PE	Alexa Fluor® 647	APC
**Biomarker**	CD3	CD16	MHC II	HLA ABC	CD21	CD94
**Monoclonal antibody**	Mouse anti dog CD3	Mouse anti Human CD16	Rat anti dog MHC Class II	Mouse anti Human HLA ABC	Mouse anti dog CD21	Mouse anti dog CD94
**Clone**	CA17.2A12	eBioCB16	YKIX334.2	W6/32	CA2.1D6	HP-3D9
**Company**	Bio-Rad	Invitrogen	Bio-Rad	Bio-Rad	Bio-Rad	Invitrogen

Supplementary Table1: Fluorochrome panel used to phenotype pNK cells and moDCs. Monoclonal antibodies directly conjugated, the respective clone and the emission filters used are indicated.

Gene	Primer	Fragment size (bp)	TA (ºC)	Reference
β-actin	FW-5’ACGGAGCGTGGCTACAGC3’	61	60.5	Sauter et al., 2005
	RV-5’TCCTTGATGTCACGCACGA3’			
CD80	FW-5’GCAGCAGAAGCCATGGATTAC3’	84	60	Yasunaga et al., 2003
	RV-5’CACCAAGAGCTGAGAGACCTTGA3’			
CD86	FW-5’CGAAACCCACCCCTGATG3’	70	60	Yasunaga et al., 2003
	RV-5’CACAAAATGACCAACATTACAAGCA3’			
TGF-β	FW-5’CAGAATGGCTGTCCTTTGATGTC3’	79	60	Huang, 2008
	RV-5’AGGCGAAAGCCCTCGACTT3’			
IL-10	FW-5’CAAGCCCTGTCGGAGATGAT3’	78	54	Yu et al., 2010
	RV-5’CTTGATGTCTGGGTCGTGGTT3’			
IL-12p40	FW-5’CAGCAGAGAGGGTCAGAGTGG3’	109	58	Peters et al., 2005
	RV-5’ACGACCTCGATGGGTAGGC3’			
IL-13	FW-5’CCCAGTGACTCGGGTTTAGA3’	116	60	*
	RV-5’GCAGCATCTCTGACCCTTTC3’			
CCL3 (MIP-1α)	FW-5’CCAGGTCTTCTCTGCACCAT 3’	84	59	*
	RV-5’AACTTGCGTGGAATCTGCTT3’			
CCL4 (MIP-1β)	FW-5’TCCTACTGCCTGCTGCTT3’	76	58	Menezes-Souza et al., 2012
	RV-5’GCTGGTCTCAAAGTAATCTGC3’			
CXCL8 (IL-8)	FW-5’CACTCCACACCTTTCCATCC3’	120	60	Harman et al., 2014
	RV-5’GTCCAGGCACACCTCATTTC3’			

Supplementary Table 2: Primers for amplification of cytokines, chemokines, and costimulatory molecules mRNA. List of forward (FW) and reverse (RV) primers, base-pair (bp) number of amplified fragments, and primer annealing temperature (TAN) for each studied gene. * Indicates the primers designed by the authors with Primer3 software 4.1.0

Product	Vendor	Catalog #	Concentration
Schneider's Insect medium	VWR	BWSTL0207-500	
Fetal Bovine Serum, Heat Inactivated	VWR	10802-772	
Penicillin/Streptomycin	Biochrom	A 2213B	10.000 U/10.000 µg/ml
Exosome-Depleted FBS	Thermo fisher	A2720803	10% (v/v)
Phosphate Buffered Saline (PBS)	VWR	97062-732	10×
Roswell Park Memorial Institute medium 1640 (RPMI)	VWR	392-0426P	
Recombinant canine IL-4	R&D Systems	754-CL-025/CF	
Hystopaque® 1077	VWR	ICNA021908378 0	
NK Cell Isolation Kit microbeads	Miltenyi Biotec	130-092-657	
Ethylenediaminetetraacetic acid (EDTA)	Sigma	E8008	2 mM
NK Cell Biotin-Antibody Cocktail	Miltenyi Biotec	130-092-657	
MS buffer	Miltenyi Biotec	130-092-657	
NK Cell MicroBead Cocktail	Miltenyi Biotec	130-092-657	
MS columns	Miltenyi Biotec	130-042-201	
FICT-conjugated mouse anti-dog CD3	Bio-Rad	MCA1774F	
Alexa Fluor® 647-conjugated mouse anti-canine CD21	Bio-Rad	MCA1781A647	
APC-conjugated mouse anti-dog CD94 monoclonal antibody	Invitrogen, Thermofisher	17-5094-42	
FICT-conjugated Human CD16 monoclonal antibody	Invitrogen, Thermofisher	16-0167-82	
FICT-conjugated rat anti-dog MHC class II monomorphic	Bio-Rad	MCA1044F	
Paraformaldehyde	Sigma	30525-89-4	2%
NZY Total RNA Isolation kit	NZYTech	MB13403	
NZY First-strand cDNA Synthesis Kit	NZYTech	MB12502	
Mouse anti-human HLA ABC	Bio-Rad	MCA81PE	
TACSTM Annexin V FITC	R&D Systems	4830-250-K	
Propidium iodide	Thermo fisher	P3566	
Dog/canine perforin 1 PRF1 ELISA Kit	Novatein Bioscience	BG-CAN11855	
Human granzyme B ELISA Kit	Quimigen	EA100653	

Supplementary Table 3: List of reagents and kits used in this study, their vendors, catalog number and concentration
